# The Role of Music in Families of Children With Hearing Loss and Normal Hearing in Australia, Finland, and the UK

**DOI:** 10.3389/fnins.2019.01002

**Published:** 2019-09-26

**Authors:** Valerie Looi, Ritva Torppa, Tania Prvan, Debi Vickers

**Affiliations:** ^1^SCIC – An RIDBC Service, Sydney, NSW, Australia; ^2^Advanced Bionics (Asia Pacific), Sydney, NSW, Australia; ^3^Logopedics and Cognitive Brain Research Unit, Department of Psychology and Logopedics, Faculty of Medicine, University of Helsinki, Helsinki, Finland; ^4^Department of Mathematics and Statistics, Macquarie University, Sydney, NSW, Australia; ^5^Department of Clinical Neurosciences, The University of Cambridge, Cambridge, United Kingdom

**Keywords:** music, children, home environment, upbringing, hearing loss, family, culture

## Abstract

The primary aim of this current study was to compare the role, importance and value placed on music by families with normally hearing (NH) children, to those who had a child with a hearing loss (HL) who wore either hearing aids and/or cochlear implants. A secondary aim was to see whether this differed between the countries. Parents of children aged 2–6 years living in Australia, Finland, and the United Kingdom were invited to complete the Role of Music in Families Questionnaire (RMFQ). Two groups of participants were recruited from each country: (i) parents of NH children, and (ii) parents of children with a HL. The RMFQ had seven subsections covering topics such as music participation, attitudes to music, importance of music in the family, and future perspectives on music. Three hundred and twenty-two families of NH children, and 56 families of children with HL completed the questionnaire (Australia: 50 NH, 25 HL; Finland: 242 NH, 21 HL; United Kingdom: 30 NH, 10 HL). Analyses compared between NH and HL groups within each country, and between the three countries for the NH group, and the HL group, independently. Overall, there were few significant differences between the participation levels, role, or importance of music in families with NH children compared to those with a child who had a HL, regardless of whether the families lived in Australia, Finland or the United Kingdom. Children first started to respond to music at similar ages, and overall music participation frequency, and music enjoyment were relatively similar. The importance of music in the family was also similar between the NH and HL groups. In comparing between the countries, Finnish children had a tendency to have higher participation rates in musical activities, with few other differences noted. Overall, the results of this study indicate that children, regardless of hearing levels or country of residence, have similar levels of music engagement and enjoyment, and HL is not seen as a contraindication to music participation and involvement by the parents involved in this study.

## Introduction

Music is pervasive, transcends cultures and spoken language, and plays a multitude of different roles across the life span. For a baby or infant, singing can soothe, comfort, calm or entertain (Custodero, 2006; Ilari et al., 2011). As the baby progresses through infancy to becoming a toddler, music can also enhance language development, parental and social bonding, and musical development (Trehub, 2001; Costa-Giomi and Ilari, 2014; Virtala and Partanen, 2018). Parent–child musical activities include not only joint singing of songs, but also playing instruments, dancing, listening to music, and spontaneously making up new music or songs (Barrett, 2011). Williams et al. (2015) showed that higher regularity of these shared home musical activities was associated with children having better vocabulary, numeracy, attentional and emotional regulation, and prosocial skills.

This contribution of music to a child’s upbringing and to family life seems to transcend culture and country. Ilari (2013) describes a qualitative study where unstructured interviews were conducted with 13 families of 7-year old children in nine countries (Greece, Netherlands, Denmark, England, Spain, Kenya, Taiwan, Israel, and the United States). These interviews comprised of 1–2 h home visits, where parents and children were asked to talk about their child’s music participation and experiences, used in collation with photographic and text descriptions of the home, location, and musical resources in the household. Thematic analyses were conducted. Although there was no between-country comparisons made, the authors reported that all 13 families had stated that music was important in their children’s lives, and that participation in organized music activities provided their child with an opportunity to discover, enjoy, and hopefully love music.

In another qualitative, natural observation study, Young and Gillen (2007) videoed a single day of home-care for seven children, aged 2.5 years, each one from a different country (Canada, Italy, Peru, Thailand, Turkey, United Kingdom, and United States). The videos were then analyzed to look for music-related activities that occurred during the day such as singing as part of the caregiver–child interaction, music that was turned on (e.g., CD) during the day, dancing, to name just a few. The authors provide a qualitative description of the musical activities observed for each child in each country, and speculate as to the potential socio-cultural factors that might have impacted on this, and how music participation is influenced by local social and cultural considerations.

There is a growing body of evidence on the associations between participation in more musical activities and improved speech perception and language skills for normally hearing (NH) listeners. Trained musicians have an increased ability to selectively engage and sustain their auditory attention, a finding referred to by some as the ‘musician’s advantage’ (Moreno and Bidelman, 2014). This cognitive advantage may subsequently transfer from music specific skills to other categories of perception and executive functions (Schellenberg, 2004). A recent systematic review of the literature by Coffey et al. (2017) investigated the notion that music training could improve speech in noise perception for NH adults. The review reported little consistency amongst the studies in determining one mechanism behind the musician advantage, yet 18 of the 20 studies reviewed did support the existence of an advantage for musicians in speech in noise perception.

To shift the focus to children, a longitudinal study measuring children’s speech in noise perception ability with music training was conducted by Slater et al. (2015). Forty six NH children (mean age of 8 years, *SD* = 0.72) were involved in the 3 years study, where they were randomly assigned to one of two groups. The first group commenced 2 years of music training (2–4 h a week) straight away, whilst the second group waited a year, and subsequently received 1 year of training. There were 38 children included in the final analyses, 19 per group, with a significant improvement for speech in noise perception being seen for the group who received 2 years of music training (*p* = 0.001). Slater et al. (2015) attributed the observed improvement to a ‘musician’s advantage’ resulting from specific training programs, not prior musical experiences. Musical training is associated with better pre-attentive processing of speech sounds of a foreign language (Intartaglia et al., 2017) and better word learning (Dittinger et al., 2017). There are several randomized controlled trials for children with NH, and these have shown that when compared to other training, music training (especially when including singing) enhances non-musical skills including reading skills, phonological awareness (Dege and Schwarzer, 2011; Flaugnacco et al., 2015; Gordon et al., 2015; Patscheke et al., 2018), speech segmentation (Francois et al., 2013), executive function (Jaschke et al., 2018), and verbal intelligence (Jaschke et al., 2018; Linnavalli et al., 2018). The possible benefits of music training have also been shown in brain imaging studies. For example, gray matter volume in areas involved in auditory processing increases with more musical training (e.g., Schneider et al., 2002; Gaser and Schlaug, 2003), as does connectivity between frontal and auditory areas (Halwani et al., 2011; Dittinger et al., 2018; Oechslin et al., 2018).

Adolescents and adults with NH regulate their mood with music, and music has emotional, social, and psychological roles in our lives (Saarikallio and Erkkilä, 2007; Saarikallio, 2011). Hence, as music is important and beneficial through the lifespan, it is important to introduce it early into a child’s life. Denac (2008) reports that early positive experiences with music will influence the child’s formation of their general attitude toward musical culture and engagement, and subsequently their interest and participation in musical activities. The rate of development of a child’s musical abilities is strongly linked to their early experiences of music before entering school, in which parents/caregivers and early childhood educators play a significant role (Denac, 2008).

Although the above studies have focused on NH listeners, there is no reason to believe that the benefits of music do not extend to individuals with a hearing loss (HL). The possible benefits of music and musical activities have already been recognized in the habilitation of children with HL. Playing musical instruments and singing is often used in speech and language therapy to engage them into the world of sounds, keep them attentive, and enhance their auditory perception (Estabrooks, 1994; Ronkainen, 2011). A longitudinal study by Good et al. (2017) compared music to art lessons for 25 CI recipients (aged 6–15 years; mean = 10 years), for music and emotional prosody perception. Music training consisted of piano lessons, music theory and singing songs. The music lesson group showed significant improvements in music perception and emotional prosody discrimination at both the mid-point (3 months) and end-point (6 months), with no improvements for the children receiving art lessons. Torppa et al. (2014a) found that children with CIs who were reported to sing more at home post-implantation were more sensitive to changes in musical pitch and timbre as assessed with attention-related brain responses. Furthermore, Torppa et al. (2014b) administered a questionnaire to children with CIs, divided into two groups: (i) those having more involvement in formal and informal musical activities (active music engagement at home and outside of the home), (ii) and those having less music engagement, and assessed their performance twice (follow-up period approximately 16 months). Greater levels of active music engagement was strongly linked to better perception of the fundamental frequency (pitch), intensity, and prosodic stress in the speech stimuli, along with better development of auditory working memory. Additionally, better pitch and intensity perception was associated with better perception of speech stress. At the end of follow-up period, musically active children with CIs were better in word finding, verbal intelligence and phonological awareness, than less musically active children, with higher levels of parental singing being associated with better word finding and verbal intelligence (Torppa et al., 2019).

Gfeller et al. (2019) recently published retrospective data on a cohort of 76 pediatric CI users where they extracted pitch perception (pitch ranking of piano tones) test scores, as well as responses to two questionnaires – one on familial music engagement both whilst growing up, as well as ‘currently’ at the time of the study (providing a ‘familial engagement in music score’), and the second on formal music involvement in classes and ensembles whilst growing up (providing a ‘music engagement’ score, as well as a sub-score on the duration of these music classes over time, quantified in years). They found that better pitch perception correlated with the overall ‘music engagement’ score, as well as the duration of time they were involved in these music classes. Interestingly, pitch perception was a significant predictor for the speech test scores (Gfeller et al., 2019). Musical training has also been shown to improve both music perception and music enjoyment for adult CI and HA users (Looi et al., 2012a,b). Overall, evidence indicates that for children and adults with HL, musical training is associated with improvements in not just music perception and music enjoyment, but also language and non-musical auditory perception skills.

Given the potential that music involvement offers to children, regardless of their hearing levels, it is important to ascertain whether parents of children with HL value music engagement in the same ways that parents of children with NH do. That is, do parents of children with HL de-prioritize music involvement or consider their child’s HL as a contraindication to music involvement? This is important as it could potentially result in children with HL having less exposure to music, reduced opportunities to benefit from music participation, and/or lower music enjoyment levels compared to children with NH. Positively, there is some preliminary evidence showing that family values and priorities are more related to a child’s music involvement and exposure than hearing-related factors. A study conducted by Driscoll et al. (2015) involved parents of 32 families who had a child with a CI, with 28 of these families also having another child who had NH. Parents were asked to complete a survey regarding their children’s music participation and the impact of family values on musical engagement. Children were attending either preschool or primary school, with a mean age of 9.88 years (*SD* = 1.36). Correlations were performed between the parent’s ratings of importance, the child’s hearing ability, and their level of musical involvement. Results of this study revealed that CI and NH siblings from the same families had similar levels of musical involvement, with little difference in frequency of engagement or participation in formal lessons. That is, regardless of hearing status, children from the same family had music participation and enjoyment levels that reflected their parent’s values. Importantly, there was no significant association between hearing status and musical involvement; it was the values of the parents that was the dominant factor determining if a child actively engaged with music, highlighting the significant impact parent attitudes play in children’s music involvement (Driscoll et al., 2015). In line with this, the retrospective analyses by Gfeller et al. (2019) showed that current familial engagement in music was predictive of the Music Engagement Score, but age or time with the CI was not associated with music engagement.

One question that arises as a result of the Driscoll et al. (2015) study is whether the fact that the parents had a child with a CI impacted on their overall attitudes. In other words, did parents consciously (or subconsciously) change their attitude to music and the role of music in their children’s lives, to compensate for their child with a CI; if their children all had NH, would attitudes or expectations have differed? Hence the main aim of this current study was to compare the role, importance and value placed on music by families who had only NH children, to those who had a child with a hearing impairment (HI) and wore either hearing aids (HAs) and/or CIs. That is, is there a difference in the role of music, and attitudes to music between families of children with NH compared to families who have a child with a HL. Is there a difference between the children’s engagement with music, participation in music, or enjoyment of music? A secondary aim was to see whether this differed between three different countries – i.e., are there cultural considerations that need to be considered?

## Materials and Methods

This study was conducted in Australia, Finland, and the United Kingdom (UK), with appropriate institutional ethics approvals being obtained for all countries.

### Participants

Parents of children aged between 2 to 6 years were invited to complete the questionnaire. There were two groups of participants for each country – families with NH children (NH group), and families who had a child with a HL (HL group). Children in the HL group could use either hearing aids (HAs) and/or CIs, fitted unilaterally, bilaterally or bimodally (CI in one ear, HA in the contralateral ear), but had to have been fitted with their first hearing device at age two or younger. Families of children with additional disabilities and families of NH children who had a sibling with a HL were excluded from the study. As this was an anonymous online survey, it was not possible to pre-screen individuals against the inclusion and exclusion criteria. Participants had to click they had read and agreed to the online Participant Information Consent Form statement before proceeding, and that they met the study criteria.

### Materials

The Role of Music in Families Questionnaire (RMFQ), initially developed by Looi et al. (2018) was furthered for this study. There were three versions of this questionnaire, one for parents of children with NH, one for parents of children fitted with HA(s), and one for families of children fitted with CI(s). It had seven subsections, which covered the topics of: A-General Information, B-Childhood Music Participation and Experiences, C-Attitudes and Reactions to Music, D-Music Resources, E-Overall Importance of Music in the Household and Family, F-Music Listening Preferences and G-Future Perspectives. The survey comprised both closed- and open-ended questions, taking an estimated 30–45 min to complete. For Finland, an extra section H was added covering questions related to speech production, singing, and factors that encourage singing, however, these will not be covered in this paper. The RMFQ was broadly adapted from the Music Engagement Questionnaire: preschool and elementary (MEQ-P/E) used by Driscoll et al. (2015), with several major changes. Firstly, the RMFQ extended beyond the MEQ-P/E by having three separate surveys for the three sub-groups of families, and included children with HAs. The MEQ-P/E had a total of 26 questions, while the RMFQ had a total of 82 questions covering a broader range of topics. The target age group for the RMFQ was children aged 2–6 years, as opposed to the MEQ-P/E which included children up to 12 years of age. Finally, the MEQ-P/E was designed for families who had one child with a CI, and one child with NH. That is, comparisons were intra-family, rather than between-families as in the current study.

### Procedure

The three versions of the RMFQ were initially pilot tested with five families in Australia to ensure it was clear, response options were valid, and questions were interpreted as expected. The questionnaires were then translated for Finland, with country-specific adaptations and pilot testing being conducted in Finland and the UK. The adaptations were predominantly in the first section, with response categories to demographic questions being changed to suit the country (e.g., the income ranges, education categories etc.). The RMFQ was then uploaded onto an online survey portal. In Australia and Finland, Qualtrics^1^ was utilized. In the UK, University College of London web-based, secure survey tool “Opinio” was used (UCL, 2019)^2^. The questionnaire could be completed on a computer, tablet and/or smart phone, and no personal identifying information was collected.

In all countries, flyers containing the study information and questionnaire links were distributed via a number of hearing-related organizations, clinics, and charities. Data collection time varied slightly between the three countries, but was approximately 3 months.

### Data Analysis

Data from the HA and CI questionnaires were grouped together to form a HL group, with analyses predominantly focusing on comparing this group to the NH group. It should be noted that Section B included questions related to the frequency of participation in, and enjoyment of, various musical activities. These were scored with different scales between countries. Frequency was scored on a 7-point scale for Australia and Finland: 0 = don’t know, 1 = less than monthly; 2 = once a month; 3 = 2–3 times a month; 4 = once a week; 5 = 2–3 times a week; 6 = 4–6 times a week; 7 = daily. For the UK, a 6-point scale was used: 0 = don’t know, 1 = less than monthly; 2 = once a month; 3 = 2–3 times a month; 4 = once a week; 5 = 2–6 times a week; 6 = daily. Hence for analyses, a 6-point scale was used, with responses in categories ‘5’ and ‘6’ from Australian and Finnish respondents being combined. Enjoyment was scored on a scale from 1 to 10 (1 = Does not Enjoy; 10 = Very much enjoys). Data was analyzed to assess for differences within each country for the effect of a HL (i.e., NH vs. HL for the same country), as well as between the three countries (i.e., Australia vs. Finland vs. UK) for (i) NH families and (ii) HL families.

Based on the research questions, statistical comparisons were predominantly made: (1) between countries, separately for NH and HI children; (2) between NH and HI children, only within each of the three countries (but not between countries; i.e., for example, no comparisons were made between NH children from one country to HI children from a different country).

As the exact statistical test used varied for the different sections due to the different types of data and different comparisons made, details of the test(s) used will be provided in the Results section under the applicable subsection. In general, for most of the statistical comparisons, Mood’s Median tests were used as distributions of the data did not have the same shape (an assumption required for tests such as the Mann–Whitney *U*, or Kruskal–Wallis tests). The assumptions for parametric tests such as t-tests and/or Analyses of Variance (ANOVA) were not met on most occasions. Comparisons between proportions were made using Chi-square tests, or Fisher’s Exact test when the assumptions for the Chi-square test failed (i.e., if more than 20% of cells had an expected count below 5; the Chi-Square tests needs this to be no more than 20% for the results to be valid). Bonferroni corrections were made for all multiple comparisons. Correlational analyses were performed using Spearman’s Rank Correlations, as the bivariate normality assumption was violated. As is inherent to questionnaires, the number of respondents differed for some of the questions, and where applicable, the number of respondents who answered a particular question is provided in the tables provided. Analyses were performed using either SPSS version 23.0 and/or Minitab version 17.0.

## Results

In total across the three countries there were 322 families of NH children, and 56 families of children with HL. For Australia, there were 50 NH children (29 Male, 21 Female) with a mean age of 4.00 years (*SD* 1.102), and 25 hearing impaired (HI) children (15 Male, 10 Female) with a mean age of 3.54 years (*SD* 0.603). The NH children were significantly older than the HI children in Australia (*p* = 0.022; two-sample *t*-test). For Finland, the mean age of the 242 NH children (109 Male, 132 Female) was 3.92 years (*SD* 1.195), with the mean age of the 21 HI children (6 Male, 15 Female) being 3.42 years (*SD* 1.257). The UK cohort comprised 30 NH children (15 Male, 14 Female, 1 no response) with a mean age of 4.17 years (*SD* 1.341), and 10 HI children (5 Male, 5 Female), mean age 3.95 years (*SD* 1.252). There was no statistically significant difference between the ages of the NH and HI children in Finland, or the UK (Mood’s Median Tests). There was also no statistically significant difference between the ages of the NH children across the three countries, or the ages of the HI children across the three countries (Mood’s Median Tests).

### Section A – General Information

This section covered general demographic and hearing-related information. Combined across the NH and HL groups, 92% of the surveys were completed by the mother in Australia, 99% in Finland and 95% in the UK. For Australia, 91% of the respondents spoke English as their main language at home, with 99% of the children being born in Australia, and 100% brought up in Australia. Eighty percent identified their culture as ‘Australian.’ For Finland, 98% of respondents spoke Finnish as their main language, 99.6% were brought up in Finland, and 96% identified their culture as ‘Finnish.’ For the UK, 70% of the respondents spoke English as their main home language, 85% were brought up in the UK, and 65% of them identified their culture as ‘British.’ For Australia, the most typical maternal education level was a Bachelor degree (33%), with 4% at the highest educational level (Ph.D. or Doctorate). Finnish results were very similar to Australia, with the most typical maternal education level being a Bachelor degree (38%), and 4% at the highest educational level (Ph.D. or Doctorate). For the UK 48% of respondents had a Bachelor Degree and 7.5% were educated to Ph.D./Doctoral level.

For the children in the HL group, the mean age diagnosed with HL was in Australia 0.39 years (*SD* 0.909), 0.71 years (*SD* 1.017) in Finland, and 0.78 years (*SD* 1.57) in the UK. The UK HL cohort included two children with progressive hearing losses, hence the large standard deviation. The level of HL the child was diagnosed with was most typically moderate in the right ear (28%) and moderately severe in the left ear (24%) for Australia. In Finland, the majority of children had a profound loss (both ears) (57%), with 29% having bilateral moderate losses. In the UK, the cohort contained three children (30%) with moderate bilateral losses, one child with a bilateral moderately severe loss, five children with bilateral severe to profound losses, and one child with a slightly asymmetric losses with the poorer ear being moderately severe. For Finland and the UK, all HA users (Finland, *N* = 7; UK, *N* = 4) and all CI users (Finland, *N* = 14; UK, *N* = 6) were fitted bilaterally. In Australia, of the 17 HA users, 13 were bilaterally aided, 4 unilaterally aided. Seven of the eight CI children were bilaterally implanted, with the other using bimodal stimulation. For the CI recipients, the mean age at of implantation was 1.45 years (*SD* 0.960) in Australia, 0.85 years (*SD* 0.245) in Finland, and 2.33 years (*SD* 1.212) for the UK. For the HA users, the mean age of first HA fitting was 0.87 years (*SD* 1.263) in Australia, 1.12 years (*SD* 1.046) for Finland, and 1.18 years (*SD* 1.891) for the UK. The mean time with their respective hearing device, in years, was: Australia, CI, 2.20 (*SD* 1.336), HA, 2.42 (*SD* 0.955); Finland, CI, 2.42 (*SD* 1.318), HA, 2.11 (1.318); UK; CI, 1.92 (*SD* 1.531), HA, 2.92 (*SD* 1.215).

### Section B – Childhood Music Participation and Experiences

This section asked about the child’s engagement in a wide range of music-based activities. Responses to question B1, “At what age did your child first start paying attention to music?” indicated that Australian NH children first attended to music at an average age of 0.62 years (*SD* = 0.89) while the 22 HI responses indicated an average age of 0.81 years (*SD* = 0.089), with no significant difference between the two groups (Mood’s Median Test).

Question B5 asked parents “How often did you sing in front of your child (face to face) during the last year?” This was followed for the NH group by the question, “How often did you sing in front of your child (face to face) during the first year of his/her life, and for the HI group (Australia and Finland only)” – “How often did you sing in front of your child (face to face) in the first year after they received their CI/HA(s)?” The scales and results are displayed in Table 1.

**TABLE 1:** Mean of responses to questions B5 and B6 for parental singing.

**Question**	**Australia**		**Finland**		**UK**	
	**NH**	**HL**	**NH**	**HL**	**NH**	**HL**
B5	5.1 (1.3)	5.3 (0.96)	5.4 (0.95)	5.4 (1.2)	4.9 (1.5)	4.6 (1.6)
	(*n* = 48)	(*n* = 21)	(*n* = 242)	(*n* = 21)	(*n* = 30)	(*n* = 10)
B6	5.7 (0.57)	5.6 (0.59)	5.6 (0.66)	5.5 (0.51)	5.6 (1.1)	Was not
	(*n* = 48)	(*n* = 21)	(*n* = 242)	(*n* = 21)	(*n* = 30)	asked

*6 = Daily, 5 = 2–6 times a week, 4 = Once a week, 3 = 2–3 times a month, 2 = once a month, 1 = Less than once a month, 0 = Don’t know.*

For the question related to the amount of singing in the last year (Q B5), for the NH families, Fisher’s Exact Test showed there was a significant difference between the three countries, with *post hoc* comparisons showing that the distribution of scores for Finland was significantly different to the UK distribution (*p* = 0.004) (see Table 2 for the crosstabs, showing the distribution of responses for both the NH and HI groups). The means and distributions indicate that parents in Finland sang more than in UK. For the families of children with HL, there was no difference between the countries. Within each country, a statistically significant difference in the distribution of parental singing of NH children compared to HI children was found for Finland only. Compared to Finnish NH families, it can be observed that more Finnish parents of children with HI sang ‘daily’ or ‘once a month’ for their child, while less of them sang ‘less than once a week,’ ‘2–3 times in month,’ or ‘2–6 times a week.’^3^ No other statistically significant differences were found.

**TABLE 2:** Distribution of responses for question B5.

**Response**	**Australia**		**Finland**		**UK**		**All countries**	
	**NH**	**HL**	**NH**	**HL**	**NH**	**HL**	**NH**	**HL**
	***n* (%)**	***n* (%)**	***n* (%)**	***n* (%)**	***n* (%)**	***n* (%)**	***n* (%)**	***n* (%)**
0	1 (2.1)	0	0	0	0	0	1 (0.3)	0 (0)
1	0	0	4 (1.7)	0	2 (6.7)	1 (10.0)	6 (1.9)	1 (1.9)
2	2 (4.2)	1 (4.8)	0	2 (9.5)	2 (6.7)	0	4 (1.3)	3 (5.8)
3	2 (4.2)	0	9 (3.7)	0	0	1 (10.0)	11 (3.4)	1 (1.9)
4	2 (4.2)	1 (4.8)	12 (5.0)	0	4 (13.3)	2 (20.0)	18 (5.6)	3 (5.8)
5	20 (41.7)	9 (42.9)	76 (31.4)	5 (23.8)	7 (23.3)	2 (20.0)	103 (32.2)	16 (30.8)
6	21 (43.8)	10 (47.6)	141 (58.3)	14 (66.7)	15 (50.0)	4 (40.0)	177 (55.3)	28 (53.8)

*6 = Daily, 5 = 2–6 times a week, 4 = Once a week, 3 = 2–3 times a month, 2 = once a month, 1 = Less than once a month, 0 = Don’t know. n, number of responses.*

**FIGURE 1:**
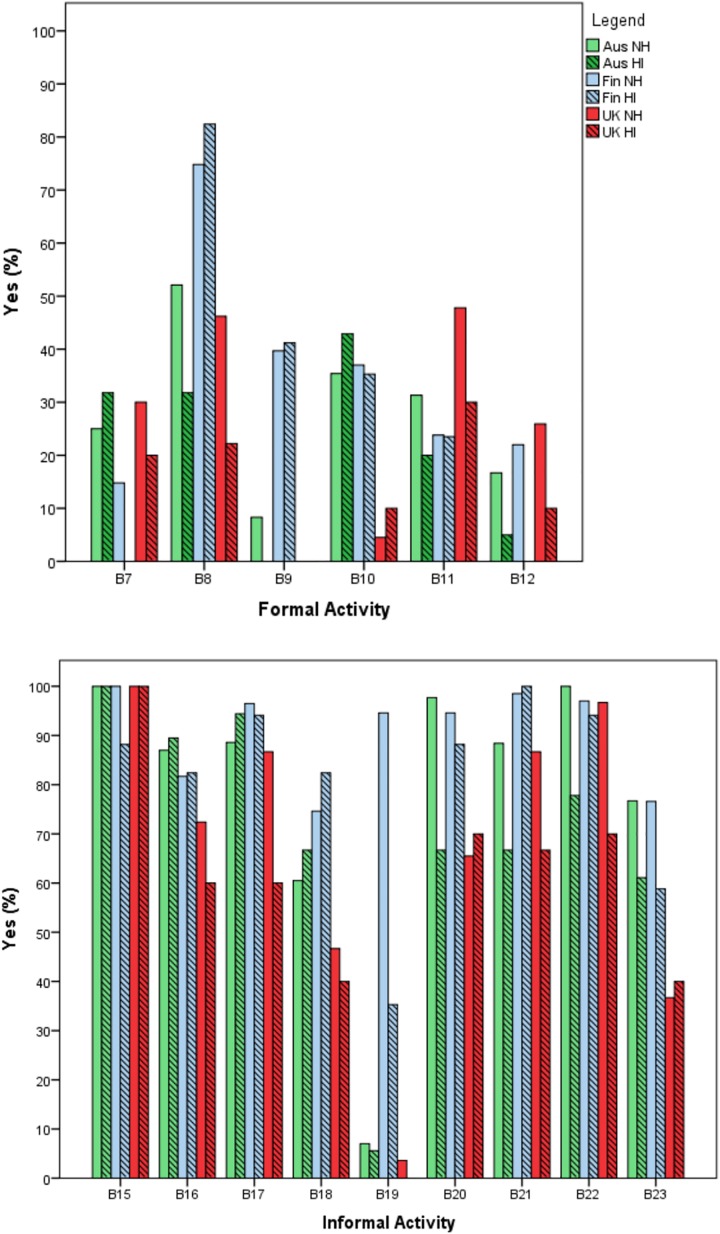
Percentage of children who had participated in each activity, for the 6 participant groups. The top panel displays the formal activities, the bottom panel displays the informal activities. Formal: B7, Music Lessons; B8, Singing Groups; B9, Instrumental Groups; B10, Special children’s music programs; B11, Dance classes; B12, Other organized music programs or activities; B13, Music classes at preschool/kindergarten/childcare; Informal: B15, Listening to music informally; B16, Social music activities; B17, Musical videos; B18, Family music activities; B19, Online music training or music games; B20, Independent music exploration; B21, Creating/making up songs or music performances for play or fun; B22, Dancing informally; B23, Live music concerts. B13’s response scale was different in Finland, and therefore is not presented in this figure.

Questions B7 – B23 covered a range of different music activities as listed in Table 3. Respondents were asked whether their child participated in each of these activities. Parents were first asked, “Has your child ever participated in this activity?” (Yes/No). The results are presented in Figure 1. If they answered yes, then the child’s frequency of participation was scored on a scale from 0 to 6, as detailed earlier in the ‘data analysis’ section, with this data used to calculate the Overall Music Participation Frequency Score (OMPFS) calculation (discussed below).

**TABLE 3:** Section B question/instrument association.

**Question**	**Activity**	**Classification**
B7	Music Lessons (formal lessons – instrument or voice)	Formal activities
B8	Singing Groups (e.g., choir)	
B9	Instrumental Groups (e.g., orchestra or band)	
B10	Special children’s music programs (e.g., Kindermusik, Yamaha, Suzuki music groups)	
B11	Dance classes (formal lessons – e.g., ballet, tap, jazz)	
B12	Other music programs or activities (e.g., those organized and run by the school, community, religious organizations etc.)	
B13	Music classes (at preschool/kindergarten/childcare)	
B15	Listening to music informally (e.g., in the car, bedtime, playtime etc.)	Informal activities
B16	Social music activities (informal, not organized activities – e.g., playing with friends)	
B17	Musical videos (TV, online, Youtube etc.)	
B18	Family music activities	
B19	Online music training or music games	
B20	Independent music exploration (e.g., playing homemade music instruments etc.)	
B21	Creating/making up songs or music performances for play or fun	
B22	Dancing informally	
B23	Live music concerts (e.g., children’s music bands, Hi-5, The Wiggles, etc.)	

*B14 asked parents, if their child was not typically involved with music, to select the applicable reasons.*

When the NH data was examined as a whole (i.e., combining the participation rates for all three countries), it was observed that all of the children participated in musical activities; for example, 100% of children listened to music informally, 97% danced, and 96% created their own songs or musical performances. In comparing between the countries, for the NH group, there were significant differences for participation (yes/no) in Singing Groups, Instrumental Groups, Special Children’s Music Programs, Dance Classes, Family Music Activities (e.g., singing or playing music together), Online Music Training Programs/Games, Independent Music Exploration, Creating Songs/Music Performances informally, and Music Concerts (Chi-square tests, or Fisher’s Exact Tests). The Finnish cohort participated significantly more (*p* < 0.005) than both the Australian and UK cohort for Singing Groups, Instrumental Groups, and Online Music Training Programs/Games. Both the Finnish and Australian cohort participated significantly more than the UK cohort (*p* < 0.005) in Special Children’s Music Programs, Independent Music Exploration, Creating/making up Music or Singing Songs informally, and Music Concerts. Finnish children participated more in Family Music Activities than UK children, but UK children participated more in Dance Classes than Finnish children (*p* < 0.005 for both). Overall for the NH children, where there was a significant difference, the Finnish children had greater participation levels, with little difference between the Australian and UK children. The UK children tended to have the lowest participation rates overall.

For the HI group, the Finnish cohort participated in Singing Groups as well as in Creating Music or Songs informally more than both the Australian and UK children. Their greater participation rates in online music training programs than the UK children was approaching significance (*p* = 0.057). Australian children had significantly higher rates of Formal Music Lessons than the Finnish children (*p* = 0.012). There were no other statistically significant differences. Overall for the HI children, less significant differences were observed than for the NH group. The within-country comparisons for participation in specific activities between NH and HI children were conducted using Fisher’s Exact Test. The only significant differences were, for Australia, for Independent Music Exploration (*p* = 0.002) and Dancing Informally (*p* = 0.005), for Finland, for Other Music Programs/Activities (*p* = 0.027) and Listening to Music Informally (*p* = 0.005), and for the UK, Dancing Informally (*p* = 0.042). In all cases, NH participation rates were higher.

#### Overall Music Participation Frequency (OMPFS)

To get an overview of the average frequency of participation in music activities, an ‘Overall Music Participation Frequency’ score (OMPFS) was calculated for each participant by averaging their frequency of participation scores from questions B7–B23, with frequency being classified on a scale from 0 to 6, as described earlier. These mean of these scores are shown in Table 4. As only activities that the child participated in were included in the calculation, the number of activities averaged differed for each child. When comparing between countries, one-way ANOVAs showed that the Finnish HI children had higher participation scores than the UK HI children (*p* = 0.011). Within each of the three countries, there was no difference between the NH and HI children.

**TABLE 4:** Mean and SD for OMPFS and OMES scores.

**Mean (*SD*)**	**Australia**		**Finland**		**UK**	
	**NH**	**HL**	**NH**	**HL**	**NH**	**HL**
	**(*n* = 37)**	**(*n* = 16)**	**(*n* = 219)**	**(*n* = 19)**	**(*n* = 30)**	**(*n* = 10)**
OMPFS (/6)	2.4 (0.63)	2.1 (0.63)	2.4 (0.83)	2.7 (0.84)	2.3 (0.74)	1.8 (0.82)
OMES (/10)	8.8 (0.91)	8.8 (1.17)	8.8 (0.95)	8.7 (1.08)	9.2 (1.21)	9.2 (1.25)

*OMPFS: 6 = Daily, 5 = 2–6 times a week, 4 = Once a week, 3 = 2–3 times a month, 2 = once a month, 1 = Less than once a month, 0 = Don’t know. n = number of responses. OMES: Scale from 1 to 10 (1 = “did not enjoy,” and 10 = “very much enjoyed”).*

#### Overall Music Enjoyment Score (OMES)

In addition to the OMPFS, an Overall Music Enjoyment Score (OMES) was calculated for each child, by averaging the ratings for the activities that the child participated in; again, the number of activities included in this calculation differed between the individual participants. The mean of these scores are shown in Table 4. Mood’s Median Tests showed that scores from the NH children in the UK were significantly higher than the NH Australian children (*p* = 0.016), and also higher than the NH Finnish children (*p* = 0.030), with no other differences. There were also no differences between any of the countries for the HI children, nor any differences between the NH and HI within each of the three countries That is, overall, NH and HI children from the same country had similar music enjoyment and participation scores.

### Section C – Childhood Music Participation and Experiences

This section asked about the child’s participation in, and reactions to, music, and was slightly different for the UK than for Australia and Finland. In Australia and Finland, the first question was only for the HI children, and asked parents whether their child’s reaction to music has changed over time since receiving their HA and/or CI. There were 36 responses in total for both countries, of which 18/36 (50%) said their child immediately became more interested in music, 4/36 (11%) said it was a gradual increase in interest in music, with 12/36 (33%) saying there was no change in their reaction. There were two ‘other’ responses. For the UK, parents of HI children were asked to rate on a scale from 1 (does not enjoy at all) to 10 (very much enjoys), whether their child enjoyed music overall. Of the 10 parents that responded, seven gave the maximum rating of ‘10’, and there was one rating of ‘7,’ and one rating of ‘4.’

**FIGURE 2:**
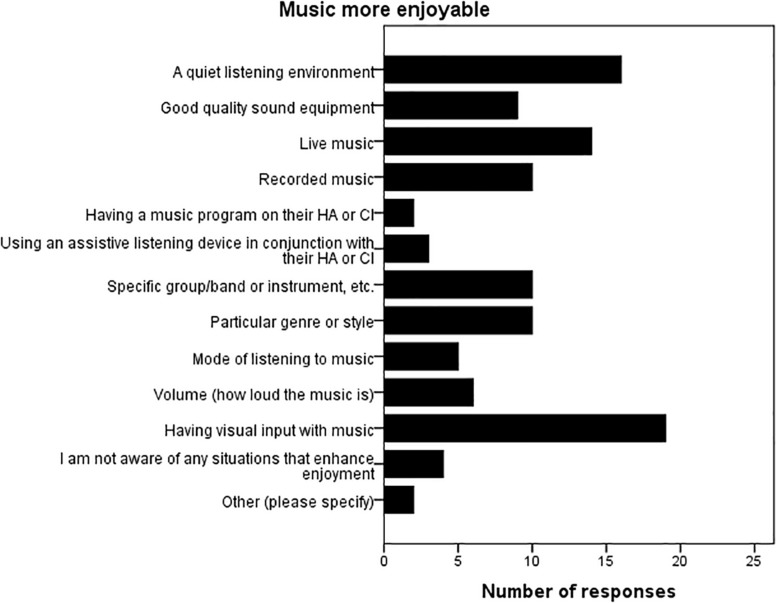
Factors that make music listening more enjoyable. Parents could select more than one factor, and the bars represent the number of time that factor was selected (Australia and Finland only; *n* = 45).

**FIGURE 3:**
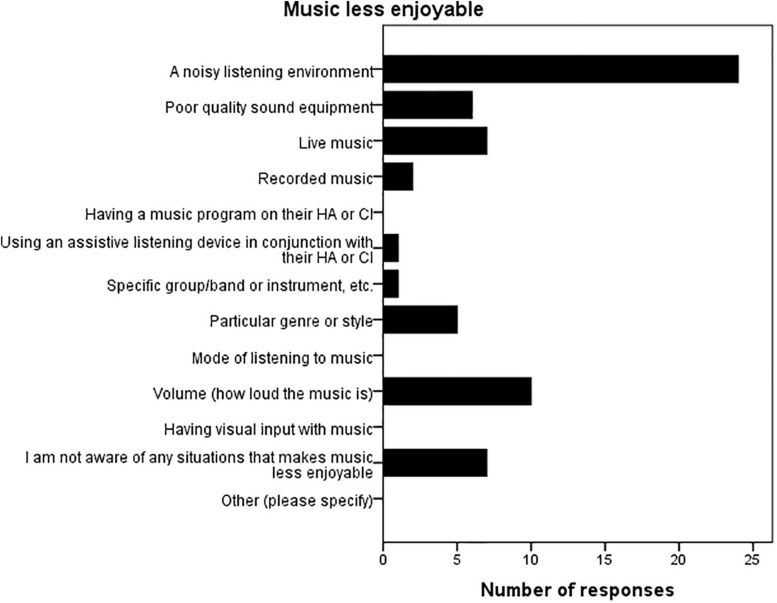
Factors that make music listening less enjoyable. Parents could select more than one factor, and the bars represent the number of time that factor was selected (Australia and Finland only; *n* = 45).

For all three countries, and all groups (including the NH group), this was followed by the question “Which of the following best describes your child’s response to music generally, in the last 6 months” with a 5-point rating scale: 1 = very much enjoys music; 2 = enjoys some aspects of music; 3 = neither enjoys nor dislikes; 4 = somewhat negative; and 5 = dislikes music. None of the NH parents selected ‘somewhat negative’ or ‘dislikes music’ and only 1 parent from the HL group rating ‘somewhat negative,’ with no ratings of ‘dislikes music.’ Statistical analyses showed no differences between the three countries nor between the NH and HI groups for the proportions who selected each option. The Australian and Finnish questionnaire for HI families then asked about factors that may make music listening more, and less enjoyable for their child with a HA or CI. Eleven different factors were listed, with respondents asked to mark all the factors that made music more enjoyable, and subsequently in the next question, all the factors that made music less enjoyable. There were also two additional options: “I am not aware of any situations that…(makes music more/less enjoyable),” and “Other (please specify).” Results are presented in Figures 2, 3. Across countries, having visual input with the music, followed by a quiet listening environment, were the two most commonly selected factors that helped to make music more enjoyable, with a noisy listening environment overwhelmingly selected as the factor that made music less enjoyable.

### Section D – Music Resources for Child

The questions in this section asked parents if they had discussed their child’s music participation with teachers, therapists or other professionals, if they had utilized music information from any of the HA or CI companies, and if they had purchased musical instruments or music resources for their child. For the NH group, only 17% of respondents said they had discussed their child’s music participation with professionals, compared to 55% for the HL group. Statistical analyses (performed with Chi-Squared Tests or Fisher’s Exact Tests) showed that for each country, the difference between the NH and HL groups was statistically significant (or approaching significance) (*p* = 0.09 for Australia; *p* < 0.001 for Finland; *p* = 0.055 for UK), with no country difference within each of these two groups.

With regard to purchasing musical instruments, 67% of the NH group and 72% of the HL group said they had purchased (or were renting, or in the process of purchasing) musical instruments for their child, with no differences within or between the countries. Ninety-two percent of the NH group and 80% of the HL group had purchased physical music resources (e.g., music books, music toys, DVD/Video, CDs) for their child. For the NH group, more Finnish parents had purchased resources than Australian (*p* = 0.011) or UK parents (*p* = 0.005). For the HL group, there was no significant country differences noted. Within each country, the difference between the NH and HL groups was only significant for the Finnish cohort (*p* = 0.007; NH higher).

### Section E – Overall Importance of Music in Your Household and Family

This section aimed to evaluate how important music was in the family and child’s life. All statistical analyses were conducted with Mood’s Median tests, with *post hoc* Chi square tests conducted where applicable. Parents were firstly asked to rate ‘music is important to our family’s life’ on a scale from 1 to 10 (1 = not at all important; 10 = very important). Results are shown in Table 5.

**TABLE 5:** Mean and SD (and ‘n’) for Section E, Section F and Section G.

**Question**	**Australia**		**Finland**		**UK**	
	**NH**	**HL**	**NH**	**HL**	**NH**	**HL**
Music is important in our family’s life	8.3 (1.54)	7.5 (2.28)	7.4 (1.63)	7.3 (1.41)	8.9 (1.47)	7.9 (3.09)
	*n* = 40	*n* = 16	*n* = 184	*n* = 19	*n* = 25	*n* = 8
Music is important in the child’s life	8.5 (1.60)	8.4 (2.02)	7.7 (1.46)	7.7 (1.53)	9.1 (1.26)	7.9 (2.71)
	*n* = 39	*n* = 16	*n* = 183	*n* = 19	*n* = 26	*n* = 9
Music is important in our other children’s lives (if applicable)	6.7 (3.59)	4.6 (3.89)	8.0 (2.16)	8.1 (1.64)	9.2 (1.39)	6.2 (2.79)
	*n* = 39	*n* = 16	*n* = 126	*n* = 11	*n* = 17	*n* = 6
My child loves music	9.0 (1.45)	8.6 (1.86)	8.7 (1.45)	8.7 (1.45)	9.4 (1.01)	9.0 (1.73)
	*n* = 37	*n* = 16	*n* = 180	*n* = 19	*n* = 22	*n* = 7
My child is good at music	6.4 (2.04)	6.0 (2.00)	8.2 (1.68)	7.4 (1.61)	7.8 (1.80)	6.8 (3.31)
	*n* = 37	*n* = 16	*n* = 180	*n* = 19	*n* = 19	*n* = 6
I think my child will be actively participating in music for the next 5 years	8.2 (1.67)	8.9 (1.34)	8.4 (1.96)	8.1 (1.91)	8.7 (1.40)	9.1 (1.46)
	*n* = 37	*n* = 16	*n* = 180	*n* = 19	*n* = 23	*n* = 8
I think my child with be actively participating in music in high school	7.2 (2.19)	7.1 (2.54)	7.3 (2.23)	6.2 (2.24)	7.9 (1.76)	8.1 (2.48)
	*n* = 37	*n* = 16	*n* = 180	*n* = 19	*n* = 23	*n* = 8
If music was optional at school, do you think your child would do it?	7.8 (1.91)	7.8 (2.30)	7.5 (2.01)	5.6 (1.57)	8.3 (2.05)	9.0 (1.92)
	*n* = 37	*n* = 16	*n* = 180	*n* = 19	*n* = 24	*n* = 7

*Mean (SD) are provided, with the ‘*n’* being the number of families who responded to that question. All scores are from 1–10; 1 being the poorest/lowest.*

Between-country comparisons for the NH group were statistically significant (*p* = 0.0004), with this difference being the UK parents rating music as significantly more important in their family’s life than the Finnish parents (*p* = 0.002). There were no other significant differences. Within each country, there was no difference between the NH and HL group.

When asked to rate how important music was in their child’s life (same scale as for ‘family life’), similar results were found with the UK parents of NH children rating music as significantly more important than Finnish parents of NH children (*p* = 0.001), with no significant country difference for the HI children, nor any significant difference between the two groups within each of the three countries individually (Table 5).

The third key question asked parents to rate the importance of music in their other children’s life (see Table 5). For the NH children, the UK parents’ ratings were significantly higher than both the Australian and Finnish parents’ ratings (Australia: *p* = 0.001; Finland: *p* = 0.010), with no country differences for the HI group. Again there were no differences between the NH and HL groups for any of the three countries.

### Section F – Child’s Music Listening Preferences

The first question of this section asked parents ‘How much music would your child actively listen to, or be involved with each week’ (in hours)? Results are displayed in Table 6. Independent Samples Median Tests showed no significant differences between NH and HI children within each country, or between the countries for the NH, or HL groups.

**TABLE 6:** Means for ‘How much music would your child listen to or be involved in each week (hrs).’

**Hours**	**Australia**		**Finland**		**UK**	
	**NH**	**HL**	**NH**	**HL**	**NH**	**HL**
	**(*n* = 28)**	**(*n* = 12)**	**(*n* = 118)**	**(*n* = 11)**	**(*n* = 17)**	**(*n* = 4)**
Mean (SD)	7.0 (7.6)	9.7 (12.4)	4.8 (5.4)	4.5 (3.8)	9.4 (8.6)	5.8 (4.2)
Median	4.0	5.0	3.0	3.0	7.0	6.5

The next two questions then asked parents to rate on a slider scale (which was subsequently converted into a number from 1 to 10 where 1 was the poorest): (i) ‘My child loves music,’ and (ii) ‘in my opinion, my child is good at music.’ As can be seen from the results shown in Table 5, overall ratings were extremely high for the first question. Mood’s Median Tests with *post hoc* Chi-Square tests (and Bonferroni corrections) were used to compare between the countries. These analyses showed that the NH Finnish ratings were significantly lower than the Australian or UK NH ratings (Australia: *p* = 0.007; UK: *p* = 0.003), with no country difference for the HL group nor any significant differences between the NH and HL groups within each country. The NH Australian ratings on whether they felt their child was good at music was significantly lower when compared to both the Finnish and UK NH children (Finland: *p* < 0.001; UK: *p* = 0.004). Again there were no differences for the HL group, or within the countries between the NH and HIL groups. There were no significant between-country differences for either the NH or HL groups in the proportion of children who would initiate age-appropriate music experiences themselves and within each country, the only difference was for Finland, with significantly more NH children doing this (Fisher’s Exact Test; *p* = 0.031).

### Section G – Future Perspectives

The first three questions of this section asked parents: (i) I think my child will be actively participating in music for the next 5 years; (ii) I think my child will be learning a musical instrument, playing in a band, or singing in a choir when they are in high school; and (iii) If music was an optional subject at primary school, do you think your child would do it? The response was made on a slider-rating scale (converted to a number between 1 and 10 where 1 was the poorer or ‘less likely’ score). Finally parents were asked whether they would support it if their child wanted to pursue music as a career (yes/no). Results for these three questions are presented in Table 5. Mood’s Median Test with *post hoc* Fisher’s Test with Bonferroni corrections (where applicable) were used for analyses of the first two questions, and Fisher’s Exact Test used for the proportional data in the last question.

There were no between, or within country differences for the first question of this section. For the second question, responses were similar between the countries for the NH children, but for the HI children, Finnish parents had significantly lower expectations than UK parents (*p* = 0.033). Within the countries, the only significant difference was for Finland with parents of NH children having significantly higher expectations than parents of HI children (*p* = 0.022). With regard to whether they thought their child would do music if it was an optional school subject, the parents of Finnish HI children had significantly lower expectations of this happening than both the Australian and UK parents (Australia: *p* = 0.009; UK: *p* = 0.001), with no difference between countries for the NH children. Within each country, only the Finnish cohort showed a significant difference between the NH and HI children (NH higher; *p* = 0.001).

For the final question, the overwhelming response was ‘yes,’ with 95% of the parents of both the NH and HI children saying they would support their child if music was their chosen career path, with no significant between or within country differences for the proportion of parents who said ‘yes.’

### Correlations

Correlational analyses were conducted to look for associations between the OMPFS or OMES and both key participant variables (age for both groups, and for the hearing impaired group, age diagnosed with HL and age fitted with device), as well as the following four questions in Sections E and F: (i) importance of music in your family’s life; (ii) importance of music in your child’s life; (iii) my child loves music; (iv) my child is good at music. For the NH group, there was a weak negative correlation between age and the OMES (Spearman’s ρ = −0.129; *p* = 0.029), with no significant correlation between age and the OMPFS. That is, older participants provided slightly lower enjoyment ratings for the activities they participated in. There were no other significant correlations for the HL group.

For the NH group, there were significant moderate correlations between both the OMES and OMPFS and all four questions listed above. For the HL group, there were significant moderate correlations between the OMES and the rating of the importance of music in the child’s life, as well as between both the OMES and the OMPFS and the ratings of ‘my child loves music’ and ‘my child is good at music.’ There were no significant correlations for either the OMES or OMPFS and the rating of how important music is in the family’s life for the HL group. These results are shown in Table 7.

**TABLE 7:** Correlations between OMPFS and OMES, and questions from Section E and F, for both NH and HI groups.

**Question**	**NH**		**HI**	
	**OMPFS**	**OMES**	**OMPFS**	**OMES**
How important is music in your family’s life?	ρ = 0.454	ρ = 0.444	Not	Not
	(*p* < 0.001)	(p < 0.001)	significant	significant
How important is music in your child’s life?	ρ = 0.504	ρ = 0.512	ρ = 0.459	Not
	(*p* < 0.001)	(*p* < 0.001)	(*p* = 0.002)	significant
‘My child loves music’	ρ = 0.459	ρ = 0.587	ρ = 0.468	ρ = 0.543
	(*p* < 0.001)	(*p* < 0.001)	(*p* = 0.002)	(*p* < 0.001)
‘My child is good at music’	ρ = 0.400	ρ = 0.399	ρ = 0.418	ρ = 0.423
	(*p* < 0.001)	(*p* < 0.001)	(*p* = 0.007)	(*p* = 0.006)

## Discussion

The major aim of the current study was to compare the role of, and attitudes to, music between families of children with NH to families with children with a HL, with a secondary aim of seeing if these results differed between three countries. The overall results showed very few differences between NH and HI children, regardless of whether the families lived in Australia, Finland or the UK, suggesting that it is the family’s attitude to music and the role it plays in the household, rather than a child’s hearing thresholds, that determines children’s involvement and engagement with music. There were few differences between the countries, either, suggesting that this finding of familial influence is fairly consistent, at least across the three countries involved in this study.

### Families of NH Children vs. Families of HI Children

Children, regardless of their hearing abilities, first started attending to music at similar ages (NH: 0.62 years; HI: 0.81 years). Briggs (1991) provides a list of music development milestones for NH children from birth to age 11. Our NH findings are relatively consistent with their developmental progression proposing that an infant will first respond to being sung to with fixed attention or cessation of movement at age 2 months, and will ‘calm’ or quieten to quiet music at around 3 months of age. Around the age of 6–8 months, the child will start to search for, and attend to, music when it is played. There has yet to be any published research on how a HL impacts on these music developmental milestones, however, our results are consistent with the proposition that these early music attention milestones would be the similar for HI children, provided they were appropriately aided or implanted at an early age.

Driscoll et al. (2015) in their cohort of 16 preschool aged American children with CIs reported that their participants first started attending to music at a somewhat older age of 1.81 years; no results for their NH children were provided nor was the age at implantation, or experience with the CI, given for children with CIs. This delay of 1 year in attending to music may be partially due to the fact that the HI children in Driscoll’s study were all CI recipients, as opposed to the mix of CI and HA users in the current study. Children with implants would have had a greater degree of HL, and would probably have received minimal auditory input during the time before they got the implant, hence the older age before they attended to music. It is also interesting to note here that in the current study, 50% of the parents in the HL group, compared to 25% of parents in Driscoll et al.’s (2015) study, reported that their child with a HL became immediately interested in music when they received their devices for hearing. The age of implantation (or initial HA fitting) was not provided by Driscoll et al. (2015), and if their cohort was implanted at a later age, it may also explain the delay in attending to music. During periods of deafness, the brain reorganizes to compensate for the lack of auditory input; once this auditory input if provided, further reorganization occurs. However, this takes time (Kral and Sharma, 2012).

To examine music engagement and participation, parents were provided with a list of 15 different music activities and asked to indicate which of these their child participated in, and the frequency of participation. Of these 15 activities, there was only a maximum of two activities where the NH cohort had a significantly higher proportion of children participating than HI children (and only one in the UK). There was no significant difference in the overall frequency of participation (as measured by the OMPFS), or enjoyment levels (as measured by the OMES), between NH and HI children. This is supported by the later question asking parents ‘How much music would your child actively listen to, or be involved with each week,’ where no significant difference was found between NH and HI children. Further demonstrating the overall high levels of music enjoyment reported in this study, when parents were asked about their child’s response to music in the last 6 months, none of the NH parents selected ‘somewhat negative’ or ‘dislikes music’ and only one parent from the HL group rated ‘somewhat negative,’ with no ratings of ‘dislikes music.’ That is, only one parent out of 308 who answered this question (combined NH and HL groups) said their child has a negative response to music, and only four parents (three from the NH group and one from the HL group) indicated a neutral reaction of ‘neither enjoys nor dislikes.’

There were no differences for any country between the proportion of NH and HI families who had procured (e.g., purchased, rented) at least one musical instrument for their child, with an overwhelming majority of parents having purchased physical music resources for their child (e.g., music books, DVDs, CDs, videos etc.). Finland was the only country where there was a significant difference between parents of NH compared to HI children; the former were significantly more likely to purchase music resources for their child.

There were also no differences between NH and HI families in rating how important music was in their family’s life, in their child’s life, or in the lives of their other children (where applicable). Overall for the NH and HI families, the mean ratings (out of 10) of as to how important music was in the family’s life, and in their child’s life were very high (NH: 7.7, 8.0; HI 8.0, 8.0, respectively out of 10), implying that music is an important part of family and upbringing, at least in these three developed Western nations. Driscoll et al. (2015), for their group of 16 children using CIs found very similar results, reporting a mean of 7.9/10, when asked to rate the importance of music in the household. No data was provided for the NH children in their study. In the current study, parents also provided similar ratings for whether their child loved music, was good at music, and if they self-initiated music, regardless of the child’s hearing status. This is consistent with the assertion that having a child with a HL does not impact on the role that music plays in the family, or a parent’s attitude to having their child engage and participate in music-related activities.

The final section of the questionnaire asked about the future, and their prediction of their child’s future involvement with music-related activities, as well as how supportive they would be of their child’s continued active engagement with music. The overwhelming response from both the NH and HL groups was that parents would support their child’s future involvement with music (95% of parents from both groups saying ‘yes’), and that they expected their child to be actively involved in music activities for the next 5 years (mean scores for all groups was greater than 8/10). All participant groups, except for the parents of Finnish HI children, had mean scores above 7/10 that their child would be learning a music instrument, playing in a band or singing in a choir, when the child was in high school. Given that all of the children in this study were aged 6 years or younger, this indicates that parent’s believe their child will continuing to participate in formal musical activities for many years to come.

### Factors That Make Music Listening More/Less Enjoyable for Children With HL

Parents were specifically asked about factors that impacted on their child’s music listening experience. For the HI children, having visual input with the music, and a quiet listening environment were the highest rated factors for improving music enjoyment, with a noisy listening environment followed by an inappropriate music volume being the most frequently rated factors that detracted from music enjoyment. Looi and She (2010) and Looi et al. (2019) asked postlingually deafened adult CI and HA users, respectively, about factors that impacted on their music listening enjoyment. In line with the present results, for both adult CI and HA users, being able to watch the performer or following the musical score/words (which are visual cues), and a quiet listening environment were also highly rated factors by the majority of adults that improved music enjoyment, with music volume (e.g., too loud/soft) and an echoey/reverberant room being highly rated factors detracting from music listening (‘noisy listening environment’ was not specifically asked in the adult studies). Collectively the results imply that factors which make music perception more difficult also reduce music enjoyment, whilst those making perception easier increase the listener’s enjoyment. Many of these factors overlap with variables that impact on speech perception, and by taking the time to proactively modify the listening environment would serve to benefit both speech, and music perception.

It is interesting to note that a special or separate music listening program was rarely selected as a factor to improve music listening for children or adults; it was selected by 2/45 parents in this study and 28/100 in the adult HA study (Looi et al., 2019). Hence it is questionable as to the extent these programs offered by device manufacturers genuinely improve music listening for the typical HI listener, or it may be that many CI or HA users are unfamiliar with the programs.

### Comparisons Between the Countries

A secondary aim of this study was to see whether there were any country/cultural differences in the role music plays in families of children in Australia, Finland and UK. To the authors’ knowledge, this has not yet been examined in existing research involving families with HI children.

#### Normally Hearing Children

Overall there were some between-country differences observed for the families with NH children in this study, particularly in the actual rates of participation in different music activities for children. However, the mean frequency of participation for the activity (as calculated with the OMPFS) between the countries was not statistically significant. Every single child listened to music, 97% danced, 96% created music performances informally. The present results fit with a Slovenian study by Denac (2008) where 176 kindergarten children (aged 5–6) were interviewed on the music activities they most preferred to participate in at home. They found that ‘listening to music’ (56%), dancing or moving to music (55%), and singing songs (48%) were the popular informal home music activities. Similarly in a Brazilian study by Ilari et al. (2011), all but one mother reported that they listened to music with their child, with 52% saying they also danced to this music with their child, and 43% saying they played along with the music whilst dancing. Our results are also in line with the findings from Williams et al. (2015) who reported that 42% of their Australian families engaged in music activities with their children 6–7 days a week, 32% using it 3–5 days a week, 23% for 1–2 days a week, and only 4% had not used music in the last week. It would seem that children, regardless of the country they live in, enjoy listening to music, and dancing to music.

In looking at the participation rates for individual activities, for all three countries, listening to music informally and dancing informally had the highest participation rates. There were some interesting observations made in comparing the participation data between the countries. Finnish children participated in singing groups, instrumental groups and online music training programs/games significantly more than either the UK or Australian children, and Finnish parents reported to spend significantly more time singing to their child in the last year than UK parents. In Finland, many parents participate with their children in or send them to ‘music play schools,’ which are common, low-cost activities, routinely provided at many daycares and usually taught by a music pedagogist (Huotilainen and Tervaniemi, 2018; Linnavalli et al., 2018). In these groups, children sing and play instruments, which may explain the high participation rates in singing and instrumental groups reported by Finnish parents.

The UK children participated significantly less in special children’s music programs, independent music exploration, creating/making up music or songs, and music concerts than either the Australian or Finnish children. It is difficult to determine the reason(s) for these differences. It may be that the availability and access to these different activities may be different across the countries. For example, one could speculate that the reduced availability or access to, and/or higher costs to attend, these special music programs in the UK may make it more challenging for parents, or that the availability and access to children’s singing groups or instrumental groups is higher in Finland (e.g., more groups available, closer to home, lower costs).

One other worthwhile finding to highlight was that the present results showed that parents sang, on average, 2–6 times a week to their children. Ilari et al. (2011) reported that 90% of the mothers in their study said they sang to their child, and half reported that others in the family (e.g., fathers, siblings etc.) also sang to the infant. This suggests that parental singing is an inherent part of a child’s upbringing, which is important given research indicating that more parental singing potentially benefits not only parent–child bonding, but also encourages children to sing and is associated with better attention, speech perception in noise and language skills (Torppa et al., 2018, 2019).

Interestingly, although the trend in music participation rates was for the Finnish cohort to have the highest proportion of children participating in many of the activities, and the UK children the lowest proportion, the Finnish parents had significantly lower ratings than UK parents when asked to rate the importance of music in their family’s life, and in their child’s life. For the latter, Finnish parents ratings were also significantly lower than Australian parents. This seems somewhat contradictory; one would expect that if a parent was willing to make the time effort to enroll their child in a large number of music activities or engage them in music making opportunities, then they must see some potential benefit or value to providing those musical opportunities. This may be related to country differences in deciding what is ‘important’ versus ‘less important’ when prioritizing different factors, or language or cultural differences in interpreting the term ‘important.’ For example, it may be that if music is a ‘routine’ or ‘expected’ part of Finnish culture and upbringing, being integrated routinely into daily life, then it is not necessarily seen by parents to be something special or a ‘priority.’ Alternatively, it may simply be that Finnish parents are more conservative when providing ratings on these kind of scales. Finnish parents also provided significantly lower ratings than Australian or UK parents as to whether their child loved music, although Australian parents provided significantly lower ratings than Finnish or UK parents as to whether their child was good at music.

For the OMES, a measure of the levels of enjoyment of the activities the children participated in, overall enjoyment scores were generally high (means ranging between 8.8 and 9.2 out of 10), with ratings from the UK NH children being higher than both the Australian and Finnish children. When asked about their child’s general response to music in the last 6 months, 99% of all the NH children had a positive response to music. Attitudes to music and the importance of music in the family unit were fairly similar across countries, and there were no country differences for any of the ‘future perspectives’ questions, for the NH children. Parents had high expectations when asked whether they thought their child would be participating in music activities for the next 5 years. When asked if they thought their child would be learning a musical instrument, playing in a band and/or singing in a choir in high school (i.e., more than 5 years from the time they were completing the questionnaire), expectations were still relatively high of this happening. An overwhelming 95% of parents said they would support their child if their child wanted to pursue music as a career. Thus, results from this cohort of NH children were similar to results from published results from other countries, suggesting that the value of music is recognized by parents globally, and children all around the world enjoy participating in, and listening to, music.

#### Hearing Impaired Children

There were even fewer between-country differences for the HI children. There was no differences in the age children started attending to music for the three countries, nor in the amounts parents sang to their child in the last year, or in the first year after they received their hearing device.

For the 15 music activities in Section B, similar to their NH counterparts, the Finnish HI children had significantly higher rates of participation in formal singing groups, as well as in making up or creating music/songs, than both the Australian and UK children. Further, only the Finnish children participated in instrumental groups – none of the Australian or UK HI children participated in these formal instrumental groups. Australian children had significantly higher rates of formal music lessons than the Finnish children, though. A similar trend seen in the NH data was observed in the HI data, in that Finnish children tended to have the highest proportion participating in most activities, and the UK the lowest proportion.

The OMPFS also showed that Finnish children participated in their chosen activities significantly more frequently than the UK children, with no difference between the Australian and Finnish, or Australian and UK children. The most commonly undertaken activity for Finnish children was creating or making up music/songs (100% of the children did this), followed by watching musical videos (94%). In contrast, for both the Australian and UK children, listening to music informally was the most commonly undertaken activity, followed by watching musical videos for Australia (94%) and independent music exploration as well as dancing informally for the UK children (70% for both). Several Finnish parents of children with HL reported that professionals such as speech therapists had informed them about the benefits of music participation and had recommended that they sing, and make music, with their child(ren), and therefore they made a conscious effort to integrate music into their daily family life. This may be one of the reasons for the country differences found for the Finnish HI children. It is also possible that music is an inherent part of more families’ lives in Finland than other countries, regardless of whether their child has NH or a HL. Kirschner and Ilari (2014) collected information on music participation and involvement for 41 Brazilian and 36 German preschool children. Half of the children in each country participated in weekly music education classes, but there were between-country differences when it came to music in the family home. Brazilian parents spent significantly more time than the German parents each day on active music making activities such as singing, or playing instruments at home with their children. The Brazilian children were also significantly more likely to sing and dance spontaneously than the German children. The authors discuss how ‘music learning’ is somewhat different between the countries, with Germany tending to be more dominated by formal music lessons or participation in an organized music group (e.g., choir, band etc.), whereas Brazilian children tended to learn to sing, dance and play music informally as part of their daily life (e.g., seeing it on TV, dancing/singing in the community etc.). The authors suggested that these findings indicate a culture-specific social learning process in learning musical conventions.

In the current study, music enjoyment levels were high, and similar between the countries. This enjoyment was also demonstrated with only one of the parents in this group amongst the whole cohort rating that their child’s response to music was ‘somewhat negative’; that is, 96% of the HI children enjoyed music. This is consistent with findings from Gfeller et al. (1998), Trehub et al. (2009), and Rocca (2012), who all report that the majority of children with HL enjoy, and benefit from, participating in music. Although research indicates that adults using CIs (Looi et al., 2007; Looi and She, 2010; Limb and Roy, 2014), or hearing aids (Chasin and Russo, 2004; Looi et al., 2007, 2018) have lower levels of enjoyment and perceptual accuracy for music compared to both NH adults as well as to when they had better levels of hearing, it must be remembered that most children are born with their HL, and/or acquire their HL at a very young age. Therefore they do not have a ‘normal hearing’ auditory template for music, but rather their memory and auditory template for music is one acquired whilst listening with their HA(s) and/or CIs. They have learnt to hear music that way, and therefore do not know any different.

There were no differences between the countries in parental ratings for how important music was in their family, or HI child’s lives. There were also no differences in parental ratings of how much their child loved music, how good they were at music, or whether they self-initiated music experiences for themselves. It is worthwhile highlighting here the high ratings for all six participant groups (NH and HI children in all countries) for ‘My child loves music,’ with all mean scores above 8.6 out of 10.

Finally, in looking toward the future, scores for whether parents thought their child would be actively participating in music for the next 5 years were high, with no difference between the countries. Finnish parents were less optimistic than UK parents as to whether their child would be participating in music in high school (i.e., learning an instrument, playing in a band or singing in a choir) and less optimistic than both UK and Australian parents as to whether their child would choose to do music at school if it was an optional subject.

Overall, there were few between-country differences for the HL cohort, and these differences were primarily in the activities the children in each country participated in. It is noted that the number of HI participants in each country was small, which would have reduced the sensitivity of the statistical testing.

### Correlations

Aside from the very weak negative correlation between age and OMES for the NH group, there were no significant correlations between either the frequency of music participation (OMPFS) or music enjoyment (OMES) and age-related participant variables (age, and for HI group, age diagnosed with HL, and age fitted with device) for either the NH and HI group. This is in contrast to the findings from LeBlanc et al. (1999) who tested 2042 students, aged 8–18, from Greece, South Korea, and the United States on their music preference, using an 18 item music listening test with Likert-type response scales. The authors looked at the factors of age, gender, and country on the music preference scores, finding a significant difference in music preference ratings between the three countries, with the variables of age and gender also contributing significantly to the ratings, and the variability in the ratings. In contrast, adult studies with CI recipients have found that neither age diagnosed with a HL or length of time with the device have been significantly correlated with music perception or enjoyment scores (Gfeller et al., 2008; Looi et al., 2012a). There are very few pediatric music perception studies involving children using CI or HAs, where correlations have been calculated to outcomes. Looi and Radford (2011) found pitch ranking scores of CI and HA users were not associated with age or duration of device use (age diagnosed with HL was not investigated), and Gfeller et al. (2019) did not find that age, age diagnosed with HL, age of implantation, or time with the CI were predictive of pitch ranking scores. The current authors could not find any hearing-impaired pediatric music appreciation studies where the researchers looked at whether participant variables were associated with the music participation or enjoyment scores.

For the NH group, both the OMPFS and OMES were moderately correlated to the importance of music for the family and child, and the parent-judged ratings of their child’s love of, and ability with, music. That is, the more important music was in the family and/or child’s life, the more often the child participated in music activities, and the more they were rated to enjoy these activities. Similarly, if parents felt their child loved music and/or were good at it, participation frequency and enjoyment was also higher. These results support the proposition that music enjoyment and participation are related to the importance of music in the familial, and individual’s life.

For the HI group, music enjoyment was higher in families who rated music as being more important in their child’s life, however, music importance in the child’s or family’s life did not associate with the OMPFS. Both the OMPFS and OMES were higher for children whose parents rated that they loved music, and/or were good at music. These correlations inherently make sense, although as is the nature of correlations, one cannot be sure if the children participate more because they enjoy it more and/or are thought to be better at it, or if it is because they enjoy it more or are better at it, which the parents had noticed, so subsequently they are provided more opportunities to participate. It is interesting to note that the importance of music in the family’s life was not significantly correlated with OMPFS, which is in contrast to the findings from Driscoll et al. (2015) for families who had a child with a CI as well as a NH sibling. This may be due to the fact that the OMPFS was a calculation of the number of times the children participated in their chosen music activities (i.e., frequency of participation), as opposed to the actual overall quantity of participation. That is, a child who participated in one activity every day would have a higher OMPFS than a child who participated in three activities once a week. Regardless, parents in this study do see music as important for their child with a HL, and provide them with regular opportunities to participate and engage with music.

### Limitations

One of the major limitations of this study was the large difference in group sizes – both between the NH and HL groups, as well as between the countries, which reduced the power of the statistical analyses, with much of the data violating assumptions for parametric testing. Additionally, there were relatively fewer HL participants than NH participants, with the HL data combining both CI and HA users.

Although the study is the first to compare between three different countries, including one non-English speaking country, all three countries are considered developed, ‘Western’ nations, all speaking a non-tonal language. Hence the cultural differences between these countries may be less than if compared to an Eastern, Latin–American, or African country. Additionally, it is acknowledged that ‘country’ and culture are different, and one cannot equate culture with nationality, or even country of residence with nationality. However, as discussed in the results for Section A, parents were also asked what ‘culture’ they identified with, with 80% of Australians, 96% Finnish, and 65% of the UK families responding ‘Australian,’ ‘Finnish’ and ‘British,’ respectively.

## Summary and Conclusion

The results of this study indicate that children, regardless of hearing levels or country of residence, have similar levels of music engagement and enjoyment. HL is not seen as a contraindication to music participation and involvement by the parents involved in this study, and families with HI children had similar attitudes and expectations of music for their child to families of NH children, with music being an important part of family life for the majority of respondents. When considered in conjunction with the findings of Driscoll et al. (2015), it could be propounded that the majority of parents do not significantly change their attitude to music or the role that music should play in a child’s upbringing, regardless of the child’s hearing thresholds. Overall, these findings are extremely positive, given the benefits that music training and participation offers to children across all facets of their development and upbringing.

## Data Availability Statement

The raw, anonymized data supporting the conclusions of this manuscript will be made available by the authors, without undue reservation, to any qualified researcher.

## Ethics Statement

The studies involving human participants were reviewed and approved by (1) The Macquarie University Human Sciences and Humanities Research Ethics Committee, Australia, (2) University of Helsinki Ethical Review Board in the Humanities and Social and Behavioural Sciences, Finland, (3) University College London Psychology and Language Sciences Ethics Board, UK. Written informed consent to participate in this study was provided by the participants’ legal guardian/next of kin.

## Author Contributions

VL conceived the study and the protocol, developed the questionnaires, oversaw the study, and led the Australian data collection. RT and DV were responsible for reviewing the questionnaires, adapting them for Finland and the UK (respectively), led their respective country’s data collection, and commented and reviewed the manuscript. TP analyzed the data and developed the figures. VL, RT, and TP compiled the tables. VL wrote the first version of the manuscript with the assistance of RT. RT was responsible for the modification of the manuscript to the final format, and submission, and is the corresponding author.

## Conflict of Interest

VL is currently employed by Advanced Bionics, a cochlear implant manufacturer. However this study was conceived, questionnaires developed, and data collection had commenced before the author started work with Advanced Bionics. Advanced Bionics have had no input into any part of this study, or this manuscript. The remaining authors declare that the research was conducted in the absence of any commercial or financial relationships that could be construed as a potential conflict of interest.

## Footnotes

1 www.qualtrics.com/uk/research-core/survey-software/

2 https://www.ucl.ac.uk/isd/services/learning-teaching/e-learning-staff/e-learning-core-tools/opinio

3 It is important to point out that the Fisher’s Exact Test compares the distribution of data, rather than the means. For example, in this case, a number was assigned to each category, so the M itself is somewhat meaningless (e.g., the M would be different if a different number was assigned to the categories. Due to the data being categorical, looking at the number of responses to each category (i.e., the distribution of the data) provides more meaningful comparisons. Hence the reason the M is the same in this case, but the results are still statistically different.
